# The role of blue light in plant stress responses: modulation through photoreceptors and antioxidant mechanisms

**DOI:** 10.3389/fpls.2025.1554281

**Published:** 2025-05-16

**Authors:** Kamel Chibani, Hussein Gherli, Mengjie Fan

**Affiliations:** ^1^ Department of Biology, Sultan Qaboos University, Muscat, Oman; ^2^ School of Life Sciences, University of Essex, Colchester, United Kingdom

**Keywords:** antioxidants, blue light, photoreceptors, ROS, stress response

## Abstract

Blue light exerts a profound influence on plant physiology by tightly regulating photosynthetic efficiency, developmental processes, and stress signaling networks. Within the photosynthetically active radiation range, blue wavelengths uniquely activate cryptochromes and phototropins, which in turn regulate processes such as chloroplast repositioning, phototropism, and transcriptional adjustments linked to stress mitigation. Under high intensity blue irradiation, photosynthetic electron transport chains and apoplastic NADPH oxidases generate reactive oxygen species (ROS), acting as key signaling intermediates yet posing oxidative challenges. Plants deploy intricate antioxidant defenses including superoxide dismutase, ascorbate peroxidase, catalase, and non-enzymatic scavengers like ascorbate, glutathione, and anthocyanins to maintain redox homeostasis and mitigate ROS damage. Emerging evidence indicates that the balance between beneficial and detrimental blue light effects is modulated by intensity, photoreceptor abundance, species-specific traits, and developmental context. This minireview explores the molecular and physiological responses to blue light, focusing on its role in stress signaling, reactive oxygen species (ROS) regulation, and antioxidant activity in plants.

## 1 Introduction

Sunlight, the fundamental energy source driving plant life and photosynthesis, extends beyond its role in photosynthesis to serve as a critical environmental cue that governs numerous aspects of plant physiology. It is composed of a broad electromagnetic spectrum, including ultraviolet (UV), infrared (IR), and visible light (VL) (Ahres et al., 2020; Pech et al., 2024). Within the visible light, blue light (400–500 nm), red light (600–700 nm) and green light (500-600nm) (Liu and van Iersel, 2021). Plants exhibit a unique interaction with light as specific wavelengths play key roles in driving their photosynthetic machinery. Historically, the photosynthetically active radiation (PAR) — the spectrum of light that facilitates photosynthesis — was delineated to span from 400 to 700 nm (McCree, 1972). Whitin this range, blue light (400–500 nm) plays an important role in photosynthesis, photomorphogenesis and plant development and nutrition (Trivellini et al., 2023). Blue light enhances photosynthetic efficiency by improving light absorption and triggering repairs mechanisms that protect against photodamage. It also plays a critical role in regulating plant growth regulation by activating specialized photoreceptors, notably cryptochromes (CRYPs) and phototropins (PHOTs), which mediate essential processes such as phototropism, stomatal opening, and chloroplast movement. (Cashmore et al., 1999; Briggs and Christie, 2002; Roeber et al., 2021). Additionally, blue light exposure provides enhances plant tolerance to frost and low temperatures by modulating the levels of key hormones, gibberellic acid (GA), salicylic acid (SA), and jasmonic acid (JA) (Ahres et al., 2023, 2025; Gulyás et al., 2025). A particular fascinating aspect of blue light signaling its dual role in the generation of reactive oxygen species (ROS). ROS function as a signaling molecules and potential sources of oxidative stress in plant cells (Jourdan et al., 2015). In plants, ROS generation is an inevitable consequence of light absorption and photosynthetic activity (Huang et al., 2019). While ROS, such as hydrogen peroxide (H_2_O_2_), superoxide anions (O_2_^-^), and singlet oxygen (¹O_2_), are normal byproducts of aerobic metabolism, blue light can enhance their production in chloroplasts, mitochondria, and peroxisomes (Banaś et al., 2012). This is particularly significant under high-intensity light, where the balance between energy capture and dissipation becomes strained, leading to overexcitation of the photosynthetic machinery (Consentino et al., 2015; Jourdan et al., 2015).

Under strong blue light irradiation, cryptochromes are activated, influencing the generation and accumulation of H_2_O_2_ at the at the plasma membrane and at the chloroplast surface (Müller and Ahmad, 2011; Consentino et al., 2015; El-Esawi et al., 2017). This response can be inhibited by ROS scavengers such as ascorbic acid, glutathione, mannitol or secondary metabolite (anthocyanin) when its added before illumination (Trivellini et al., 2023). Beside these non-enzymatic antioxidants, enzymatic antioxidants such as the peroxidase (POD), catalase, ascorbate peroxidase (APX), and superoxide dismutase (SOD) are able to attenuate the ROS generated under an excess of blue light illumination (Mastropasqua et al., 2012; Yu et al., 2017). Despite the growing body of literature on the impacts of blue light on plant physiology, many aspects remain insufficiently understood. Key questions persist: How do photoreceptors fine-tune ROS production and antioxidant defenses under varying environmental and developmental contexts? To what extent do species specific differences and genotypic variations modify blue light induced oxidative stress responses and antioxidant pathways? Therefore, in this minireview, we synthesize current knowledge regarding blue light perception, ROS generation, and antioxidant regulation in plants. Specifically, it is seeks to elucidate the relationship between blue light, receptors, and the specific antioxidant mechanisms that plants employ to cope with stress.

## 2 Blue light stress responses through photoreceptors

Plants use several classes of blue light receptors to modulate a range of physiological responses including both biotic and abiotic stresses (Roeber et al., 2021). Changes in light intensity and quality affect the plant redox machinery through ROS formation and antioxidant activity. Upon blue light exposure (390 to 500 nm), three classes of receptors are mainly modulated: cryptochromes (CRYP), phototropins (PHOT), and the members of the Zeiltupe (ZLT) family (D’Amico-Damião and Carvalho, 2018; Lin, 2000). Zeiltupe receptors are involved in setting up the circadian clock (Kim et al., 2007), whereas phototropins are plasma membrane photoreceptors that mediate phototropism, chloroplast movement, and oxidative stress responses (Demarsy et al., 2018; Rusaczonek et al., 2021).

**Figure 1:**
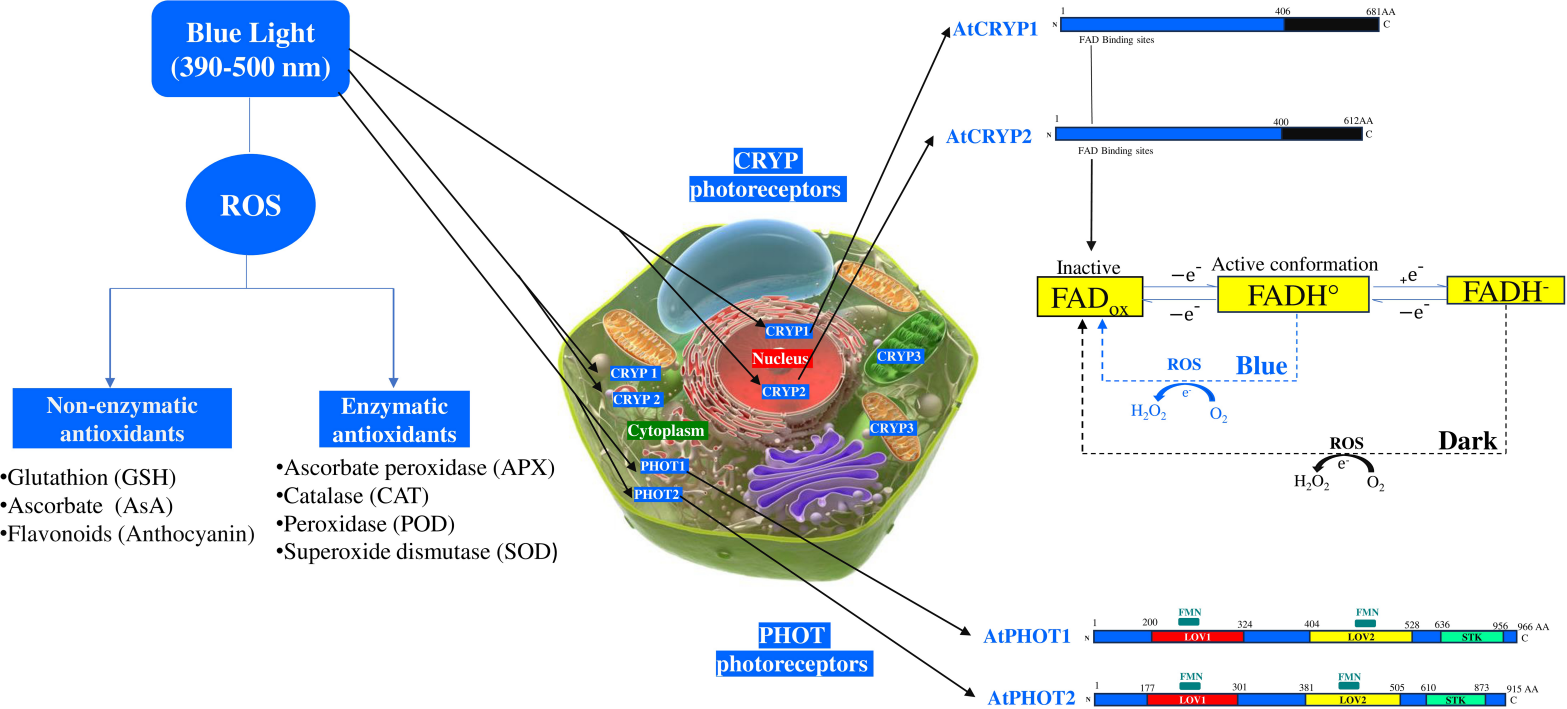
Effects of blue light on cryptochrome photoreceptors and enzymatic, non-enzymatic antioxidants. AtCRYP, *Arabidopsis thaliana* cryptochrome; AtPHOT, *Arabidopsis thaliana* phototropin; FAD_ox_, Flavin adenine dinucleotide oxidized; FADH°, Radical form of flavin adenine dinucleotide; FADH^-^, Reduced form of flavin adenine dinucleotide; FMN, Flavin mononucleotide binding domain; LOV, Light-Oxygen-Voltage-sensing domain; ROS, Reactive oxygen species; STK, Serine/Threonine Kinase domain.

Phototropins (PHOT1 and PHOT2) serve as sophisticated blue light sensors in plants. These cytosolic and plasma membrane-associated photoreceptors regulate key physiological responses, such as phototropism, chloroplast movement, and adaptation to oxidative stress. Each receptors contain two flavin mononucleotides (FMN) binding domains located within two conserved N-terminal sequences knows as LOV1 and LOV2 domains, as well as a C-terminal serine/threonine kinase (STK) domain responsible for initiating downstream signaling (**Figure 1**) (Xin et al., 2022). Light Oxygen Voltage (LOV) domains, functioning as blue light sensors, undergo autophosphorylation upon blue light photoexcitation (Christie et al., 1999; Christie 2007; Flores-Ibarra et al., 2023). These LOVs domains regulate stress responses through flavin mononucleotide-binding redox active cysteine (Yee et al., 2015). Flavin-binding proteins serve as signal receptors for redox potential, partial oxygen pressure (pO_2_), and blue light (Christie et al., 1999; Briggs and Christie, 2002; Oide et al., 2016). PHOT1 and PHOT2 proteins are structurally the same and under blue light irradiation, a portion of the PHOT1 protein is detached from the cell membrane and disperses in the cytoplasm. whereas, portion of the PHOT2 proteins can be couple to the Golgi apparatus (Wan et al., 2008; Kong and Wada, 2016). In addition, a fraction of PHOT2 has been detected on the chloroplast outer membrane (Kong et al., 2013). PHOT1 and PHOT2 are functionally distinct. While PHOT1 is activated only by low blue light intensity (< 1 μmol m^−2^ s^−1^), PHOT2 responds more effectively to high blue light intensities, ensuring efficient adaptation to varying light conditions (Sakai et al., 2001; Briggs and Christie, 2002). In Arabidopsis, *PHOT1* and *PHOT2* have partially overlapping functions and influence UV-C induced photooxidative stress responses, cell death, and photosynthesis (Rusaczonek et al., 2021). PHOT receptors induce chloroplast movement in response to blue light (Briggs et al., 2001; Wen et al., 2008). Blue light perceived by the PHOT1/PHOT2 receptors represses enhances drought tolerance by repressing the inhibitory function of the multifunctional E3 ubiquitin ligase constitutive photomorphogenic 1 (COP1) on open stomata resulting in closed stomata (Roeber et al., 2021). Arabidopsis *phot1* and *phot2* single and double mutants showed a reduced accumulation of H_2_O_2_ and more efficient photosynthetic electron transport compared to the wild type after an oxidative stress caused by UV-C treatment, whereas very little information is known about the blue light effect (Rusaczonek et al., 2021). *PHOT2* functions under high blue light intensity by preventing the damage of the chloroplast photosynthetic apparatus and contribute to high light tolerance (Kagawa et al., 2001; Roeber et al., 2021).

Cryptochromes are flavin binding blue light receptors which are involved in the growth and development of plants, photoperiodic control of flowering, and reactive oxygen species (ROS) generation (Liu et al., 2011; El-Esawi et al., 2017). These receptors, primarily absorb in the blue light region and act as key regulators of a range of biotic stress responses, such as drought, salinity, heat, and high radiation (D’Amico-Damião and Carvalho, 2018). The Arabidopsis genome possess three cryptochrome genes, *CRYP1*, *CRYP2*, and *CRYP3* which encodes their respective proteins. CRYP1 and CRYP2 are nuclear/cytoplasmic localized proteins, whereas CRYP3 is chloroplastic/mitochondrial (**Figure 1**) (Liu et al., 2011). Interestingly, nuclear CRYP2 can translocate to the cytoplasm, upon blue light exposure, suggesting dynamic spectral regulation (Jourdan et al., 2015). Whilst CRYP1 and CRYP2 are structurally similar, each containing a flavin adenine dinucleotide (FAD), but there are different in stability (**Figure 1**). The C-terminal part of the CRYP2 is shorter than CRYP1 and bears little homology (Ahmad et al., 1998). Unlike CRYP1 protein, CRYP2 is highly susceptible to degradation under various light fluences (green, UV, blue). CRY3 function as a photoreceptor in mitochondria and chloroplasts and it is part of the CRY-DASH clade of the photolyase/cryptochrome superfamily, which works to repair UV-damaged DNA in a light-dependent manner, however no study has yet confirmed their role under blue light fluence (Selby and Sancar, 2006; Mishra and Khurana, 2017). Upon blue light fluence, CRYP1 and CRYP2 are activated through interconversion of flavin redox states resulting in the production of ROS in the nucleus and potentially the cytoplasm (Bouly et al., 2007; Ahmad, 2016). The FAD, is a two-electron carrier that can be represented under different redox states or protonated forms: oxidized (FAD_ox_), semireduced (anion radical semiquinone FAD^•—^ or neutral radical FADH^•^), and fully reduced flavin (FADH^—^ or FADH_2_). Among the different redox forms, only FAD_ox_ and FAD^•—^ absorb significant amounts of blue light (~400–500nm) (Ahmad, 2016). This photon absorption is followed by an electron transfer via a tryptophan (W) pathway to excited state flavin from a proximal tryptophane residue (W) at position 397 (W397) in CRYP2 or at position 400 (W400) (in CRYP1) with the formation of FADH. Besides, the flavin-proximal W radical is turned reduced by a chain of intraprotein electron transfer through two further Trp residues W377 to W324 (CRYP1) or W374 to W321 (CRYP2) to a surface-exposed Tyr residue (Ahmed et al., 2016). Under dark condition, the flavin in the CRYP1 and CRYP2 are in inactive state (FAD_ox_), whereas under blue light illumination, it is reduced to FADH^•^ (Liu et al., 2011; Fantini and Facella, 2020). Arabidopsis CRYP1 activation by blue light results in the conversion of molecular oxygen (O_2_) to ROS (Consentino et al., 2015). Overexpressing the CRYP1 induce H_2_O_2_ formation and cell death (Müller and Ahmad, 2011; Jourdan et al., 2015).

## 3 Roles of blue light on ROS synthesis and antioxidants activity

Blue light is an enhancer of ROS formation under both normal and high light intensity and the most represented ROS are: hydrogen peroxide (H_2_O_2_), superoxide radical (O_2_^•¯^), hydroxyl radical (HO^•^), and singlet oxygen (^1^O_2_) (Borbély et al., 2022; Rahman et al., 2025). ROS are produced in almost nearly every subcellular compartment and can diffuse across the membranes. The diffusion of ROS and the types produced vary according to the light intensity/quality, plant species and the environmental conditions (Wan et al., 2008; Borbély et al., 2022). High blue light (HBL) exposure leads to ROS production through multiple mechanisms. In the chloroplast thylakoid, electron spillover from the photosynthetic electron transport chain (ETC) generates ROS (El-Esawi et al., 2017; Borbély et al., 2022). These ROS can then be converted into H_2_O_2,_ a harmful molecule that can diffuse into cytosol through chloroplast membranes (El-Esawi et al., 2017; Borbély et al., 2022). Additionally, the chlorophyll molecule itself contributes to ROS generation. During photosynthesis, if there is an excess of light energy that cannot be fully utilized or dissipated by the plant, chlorophyll can transfer excitation energy directly to oxygen, forming singlet oxygen (^1^O_2_) (Foyer and Hanke, 2022). Moreover, ROS accumulate in the cytosol after being converted to the damaging oxidant H_2_O_2_ and diffusing through chloroplast membranes. On the other hand, HBL activates the apoplastic NADPH oxidase (NOX) and the plasma membrane H^+^-ATPase enzymes, which increase the rate of O_2_^•¯^ generation and H^+^ transport across the plasma membrane (Wen et al., 2008; Xin et al., 2022). The O_2_^•¯^ generated is then converted to H_2_O_2_ by the superoxide dismutase (SOD) enzyme, which utilizes the H^+^ available in the apoplast due to H^+^-ATPase activity (Kinoshita and Shimazaki, 1999; Murakami et al., 2022). *De novo* H_2_O_2_ formed diffuses through the plasma membrane, forming a cytosolic ROS pool and alongside chloroplast-derived ROS (Borbély et al., 2022; Murakami et al., 2022). The accumulated cytosol ROS pool synthesized and the threshold of the cytosolic Ca^2+^ influences actin polymerization either directly by changing actin amino acids or indirectly by influencing the activities of actin-binding proteins (ABPs) (Rentel and Knight, 2004; Görlach et al., 2015). Actin polymerization/depolymerization enables chloroplasts to migrate along the plasma membrane to limit excessive light absorption (Xing et al., 2022).

Under blue light fluence, ROS can drive changes in the plant redox homeostasis. To cope with this harmful ROS generated under blue fluence, which leads to increased levels of singlet oxygen and hydrogen peroxide, plants increase the antioxidants biosynthesis such as the glutathione (GSH), ascorbic acid (AsA), anthocyanin and the detoxifying enzymes levels (Heyneke et al., 2013; Fantini and Facella, 2020; Rahman et al., 2025). In general, under high light intensity, the ROS generated such as the H_2_O_2_ can be scavenged in the cytosol and peroxisomes by AsA, and peroxidase (POD), in chloroplasts by AsA, GSH, and ascorbate peroxidase (APX) activity, and in vacuoles by AsA and POD (**Figure 1**) (Borbély et al., 2022). Under blue light illumination, the leaves and roots of *Rehmannia glutinosa* increased total antioxidant capacity and the activity of several antioxidant enzymes such as superoxide dismutase, catalase, ascorbate peroxidase, glutathione peroxidase (Manivannan et al., 2015). In leaves of *Camptotheca acuminata* seedlings, blue light increased H_2_O_2_, O_2_^•^¯ and stimulate the SOD and POD activities (Yu et al., 2017; Nam et al., 2018). In *Boehmeria nivea* (ramie), blue light stimulates the activities of SOD and POD (Rehman et al., 2020). Under blue light, the plant growth of *Camptotheca acuminata* seedlings was reduced, whereas SOD, POD, and catalase activities increased significantly (Yu et al., 2017). In oat leaves, enhanced APX activity was reported under blue light fluence (Mastropasqua et al., 2012). The accumulation of ROS under blue light fluence can be significantly decreased by applying green light treatment which, concurrently confers drought resistance to *Cucumis melo* seedlings (Li et al., 2024). Moreover, a combination of blue and red light significantly reduces the ROS (O_2_^•-^, H_2_O_2_) intensity in alfalfa seedlings by regulating the activity of the antioxidant enzymes such as ascorbate peroxidase, catalase, superoxide dismutase, and glutathione reductase, as well as the expression of genes related to the ascorbate-glutathione (AsA-GSH) pathway (Rahman et al., 2025) The ascorbate (AsA)/Glutathione (GSH) pathway is a major antioxidant defense mechanism that detoxifies ROS in plant cells during biotic and abiotic stress (Hasanuzzaman et al., 2019). GSH, AsA, and flavonoid (anthocyanin) are the major known non-enzymatic antioxidants triggered under blue light fluence (Toldi et al., 2019; Pech et al., 2024). H_2_O_2_ degradation is facilitated by the accumulation of the reduced forms of antioxidants such as ascorbate and glutathione in chloroplasts, peroxisomes and cytosol in Arabidopsis leaves (Heyneke et al., 2013). Under high light intensity, after the accumulation of ROS, the reduced GSH is oxidized to GSSG (oxidized glutathione) and AsA is oxidized to DHA (dehydroascorbic acid). The regeneration of the GSSG and DHA is insured, respectively, by glutathione reductase (GR) and dehydroascorbate reductase (DHAR) (Borbély et al., 2022; Zhou et al., 2023). Accumulation of the glutathione redox state varies depending on light intensity, type of species and the development stage of the plant. In wheat, the pool of GSSG is 2-fold higher in high and blue light compared to white light (Toldi et al., 2019). In young and flag wheat leaves, blue light exposure leads to a decrease in GSH content (Monostori et al., 2018). Conversely, Asghar et al. (2022) demonstrated that light intensity, especially under-far-red light, significantly enhances GSH content and freezing tolerance in two wheat genotypes. Blue light is effective in increasing AsA production in several plant species, including *Lactuca sativa*, *Spinacea oleracea*, *Brassica compestris*, and *Avena Sativa* leaves (Ohashi-Kaneko et al., 2007; Mastropasqua et al., 2012). The effectiveness of this increase depends on exposure time and light intensity (Mastropasqua et al., 2012). Under excessive blue light, ROS oxidize the AsA into DHA in lettuce leaves due to the elevated level of H_2_O_2_ (Zhou et al., 2023). The accelerated recycling or regeneration of the AsA pool can not only improve AsA levels but also decrease in the same time the ROS content in lettuce leaves (Raja et al., 2017; Zhou et al., 2023). ROS decomposition is accompanied by enhanced activity of APX, L-galactono-1,4-lactone dehydrogenase (GLDH) (AsA metabolism enzyme), and the recycling AsA enzymes such as: the dehydroascorbate reductase (DHAR) and the monodehydroascorbate reductase (MDHR) (Zhou et al., 2023). The AsA/GSH cycle and flavonoids, particularly anthocyanins, are knowns as complementary non-enzymatic antioxidant pathways involved in mitigating light induced oxidative stress (Liu et al., 2023; Foyer and Kunert, 2024). In several plant species, anthocyanins represent a crucial defense component against blue light induced (Sharma et al., 2021; Cai et al., 2023). This major class of flavonoids accumulates in the cell vacuoles and is triggered by ROS under blue light. Anthocyanins are photoprotective pigments that absorb the yellow, green and blue parts of the visible spectrum (Karageorgou and Manetas, 2006; Hughes and Smith, 2007). Under high light, anthocyanins can scavenge free radicals including DPPH· (1,1-diphenyl-2-picrylhy-drazyl, C_18_H_13_N_5_O_6_), superoxide anions and hydroxyl radicals (Zeng et al., 2009). The cross-regulation between ROS and anthocyanin production under blue light were analyzed through cryptochrome (cryp1/cryp2) knock-out genotypes of Arabidopsis, tomato and rapeseed seedlings mutants. It has been demonstrated that anthocyanins are accumulated in the cryp1 mutant, whereas no reduction in anthocyanins accumulation has been reported for cryp2 mutant (Lin, 2002; Chatterjee et al., 2006; Vandenbussche et al., 2007). Overexpressing tomato CRYP2 leads to high levels of anthocyanins in all seedling organs (Giliberto et al., 2005; Fantini and Facella, 2020), Moreover, it has been reported that under blue light, the *CRY1Pa* tomato mutant enhances the expression of bZIP transcription factor HY5 transcription factor, which acts downstream of cryptochromes and promotes anthocyanin biosynthesis (Liu et al., 2018). Blue light regulates the expression of *PHOT* genes, which encode PHOT1 and PHOT2 that play a central role in plant and algal adaptation to light excess (Sharma et al., 2021). These photoreceptors mediate photoprotective responses such as chloroplast avoidance movement, non-photochemical quenching, and carotenoid biosynthesis to avoid photooxidative damage (Li et al., 2009). While PHOT1 and PHOT2 function under low light, PHOT2 predominantly governs the chloroplast avoidance response under high blue light fluence in Arabidopsis, algae and mosses (Cazzaniga et al., 2013). *PHOT* genes contribute to photoprotection by modulating gene expression and interacting with other photoreceptors to maintain redox homeostasis and limit ROS accumulation. On other hands, Overexpressing PHOT2 protein in *Fragaria ananassa* fruits and leaves leads to an increase in anthocyanins pools and enhanced blue light stress resistance (Sharma et al., 2001).

## 4 Conclusion and future prospects

Blue light plays an important role in the growth regulation and plant development, and also participates in stress response. Understanding the complex interplay between blue light, ROS signaling, and antioxidant systems is crucial for developing strategies to improve plant stress tolerance and photosynthetic efficiency under harmful blue light conditions. One promising avenue lies in developing tailored lighting regimes that optimize blue light intensity, duration, and spectral composition to improve crop yield while minimizing excessive ROS production. Coupling these lighting solutions with a sensor-based system for real-time monitoring of ROS levels in real time could enable precision environmental management. In parallel, exploring the application of synthetic plant growth regulators or hormones that work synergistically with blue light to enhance stress resistance and yield could be a sustainable approach. Finally, alternating blue and green light, or combining red and blue light treatments can be a strategic alternative to reduce ROS formation and stimulate antioxidant production, thereby enhancing stress resistance, photosynthesis and increasing crop production. However, several key questions remain that warrant further investigation: (1) How do photoreceptors precisely calibrate ROS production and antioxidant defenses across different environmental contexts and developmental stages? (2) What molecular mechanisms underlie species-specific and genotypic variations in blue light responses? (3) How can we effectively translate our understanding of blue light signaling into practical applications for agriculture and horticulture? Further research is needed to elucidate the molecular mechanisms and genetic pathways that govern the crosstalk between blue light signaling, ROS generation, and antioxidant capacity in plants. Such insights will inform not only horticultural lighting strategies but also breeding projects and biotechnological interventions aimed at refining photoprotection and improving crop performance.
